# Repurposing Zileuton as a Depression Drug Using an AI and In Vitro Approach

**DOI:** 10.3390/molecules25092155

**Published:** 2020-05-05

**Authors:** Norwin Kubick, Marta Pajares, Ioana Enache, Gina Manda, Michel-Edwar Mickael

**Affiliations:** 1 Department of Biochemistry and Molecular Cell Biology (IBMZ), University Medical Center Hamburg-Eppendorf, Martinistraße 52, 20246 Hamburg, Germany; n.kubick@uke.de; 2 Instituto de Investigaciones Biomédicas Alberto Sols (CSIC-UAM), 28029 Madrid, Spain; mpajares@iib.uam.es; 3 Department of radiology, Victor Babes National Institute of Pathology, 99-101 Splaiul Independenței, 050096 Bucharest, Romania; ioanaenac@yahoo.com (I.E.); gina.manda@gmail.com (G.M.); 4 PM forskningscentret, 17854 Ekerö Stockholm, Sweden; 5 Neuroimmunology group, Department of experimental Genomics, Institute of Animal Breeding and Genetics, Polish Academy of Science, Postępu 36A, 05-552 Garbatka, Poland

**Keywords:** depression, macrophages, NRF2, text mining, deep neural network, AI, repurposing

## Abstract

Repurposing drugs to target M1 macrophages inflammatory response in depression constitutes a bright alternative for commonly used antidepressants. Depression is a significant type of mood disorder, where patients suffer from pathological disturbances associated with a proinflammatory M1 macrophage phenotype. Presently, the most commonly used antidepressants such as Zoloft and Citalopram can reduce inflammation, but suffer from dangerous side effects without offering specificity toward macrophages. We employed a new strategy for drug repurposing based on the integration of RNA-seq analysis and text mining using deep neural networks. Our system employs a Google semantic AI universal encoder to compute sentences embedding. Sentences similarity is calculated using a sorting function to identify drug compounds. Then sentence relevance is computed using a custom-built convolution differential network. Our system highlighted the NRF2 pathway as a critical drug target to reprogram M1 macrophage response toward an anti-inflammatory profile (M2). Using our approach, we were also able to predict that lipoxygenase inhibitor drug zileuton could modulate NRF2 pathway in vitro. Taken together, our results indicate that reorienting zileuton usage to modulate M1 macrophages could be a novel and safer therapeutic option for treating depression.

## 1. Introduction

Compound identification is one of the main bottlenecks of drug repurposing to treat depression. Drug repurposing constitutes a paradigm shift from traditional drug development approaches. Traditional drug discovery workflows suffer from high cost and long delays [1]. Developing a single new drug can cost over one billion dollars [2]. Reports estimate the duration of a traditional drug discovery cycle to be fifteen years [3]. The largest segment of time and expenses of this cycle is allocated to early development, with more than 90% of drugs failing to move beyond the first stage [4]. Repurposing of drugs that have been approved for human treatment diminishes the expenses linked with the early stages of drug development. Furthermore, this approach can reduce the delay faced by therapeutic indications. Successful examples of drug repositioning include sildenafil for pulmonary hypertension, thalidomide for erythema nodosum leprosum, and retinoic acid for promyelocytic leukemia [4]. Compound identification together with compound acquisition, development, and post-market safety monitoring constitute the main phases of the drug repurposing workflow [5]. In addition to its long duration, which lasts around two years, compound identification phase is also taunted by low success rates. Thus, enhancing the efficiency of compound identification is critical for improving drug discovery research.

The influence of M1 macrophages on the molecular pathology of depression is striking [6,7]. Data supportive of an extensive role of M1 macrophages-mediated inflammation in depression is widespread. Patients with major depressive disorder exhibit an immune profile associated with an M1 phenotype [8]. In the cerebrospinal fluid (CSF) isolated from depression patients, upregulated expression of proinflammatory cytokines produced by M1 macrophages such as TNFα, among others, have been extensively reported [9,10]. Additionally, serum IL-6 and IL-1Ra, also known to be produced by M1 macrophages, were shown to be significantly higher in major depression patients as well as in patients resistant to treatment compared to healthy controls [11]. Furthermore, analyzing peripheral blood gene expression profiles from major depression patients showed an augmented expression of genes associated with the interferon α/β signaling pathway [12]. Based on these reports, it was suggested that peripheral blood IL-1β, IL-6, TNF, and CRP could constitute reliable biomarkers of inflammation in patients with depression [13]. Moreover genome-wide association stud (GWAS) analysis showed that various proinflammatory cytokines including IL-1β, TNF, and CRP have been linked to depression and response to treatment [6]. Moreover, increased expression of various innate immune genes, including IL-1β, IL-6, TNF, TLR3, and TLR4, have been found in post-mortem brain samples from suicide victims that had depression [14]. Blocking proinflammatory cytokines such as TNF, has been shown to decrease depressive symptoms in patients with major depressive disorder [15]. In animal models as well as in vitro, M1 macrophages induced by pathogen-associated molecular patterns including peptidoglycans, lipopolysaccharide (LPS), or flagellin express inflammatory cytokines including IL-1β, IL-12, and TNFα [8]. Taken together, these observations suggest that manipulating the M1 response in major depression constitute a major drug target pathway.

Currently there are not any available drugs that specifically target the inflammation side of the major depression disorder [15]. Antidepressants (Ads) could be grouped in different pharmacological classes based on their influence on monoaminergic neurotransmission. These groups include tricyclic antidepressants (TCA), selective serotonin/serotonin and noradrenaline reuptake inhibitors (respectively, SSRI and SNRI), monoamine oxidase inhibitors (MAOIs), and vortioxetine (VORT). Various ADs have been reported to have some immunological effect. It was reported that, in depressed patients, that the plasma levels of IL-1β were significantly reduced after being treated with SSRI, SNRI, or TCA. On the other hand IL-6 levels manifested a slight decrease in patients receiving SSRI. Remarkably, there was no variation in TNF-α levels. The effect of several Ads on LPS mediated depression like behavior has also been reported. It was shown that pretreatment with amitriptyline (AMI), Escitalopram (ESC), tranylcypromine (TCP), and VORT in LPS treated mice, prevented despair-like behavior. Furthermore, TCP and VORT showed an improved brain anti-inflammatory effect, while only VORT augmented brain levels of IL-4 and reduced NF-kB expression in the hippocampus. It was also revealed that numerous ADs prevented LPS-induced hypothalamic changes and amplified plasma corticosterone. However, current antidepressants suffer from adverse side effects. TCA can cause anticholinergic, hypotensive, or sedating reactions, and are associated with impaired cognitive function [16]. SSRI and SNRI side effects include sexual dysfunction, weight gain, and sleep disturbance [17]. Patients being administrated MAOIs are at risk for an adverse hypertensive reaction, with accompanying morbidity [18]. Vortioxetine causes nausea in at least 10% of the cases [19]. Dose adjustments for Vortioxetine are required if coadministered with rifampicin or bupropion [20]. These reported supports that need for new antidepressants that specifically designed to reduce the proinflammatory profile associated with major depression disorder.

The objective of this study is to identify putative compounds that are capable of enhancing the anti-inflammatory response in depression. We utilized RNA-seq data of human macrophages treated with LPS [21]. We identified Nrf2 pathways as a putative regulator of M1 function in depression. As Nrf2 is major transcription factor regulating oxidative stress, we applied a deep learning network to search 100 previously published PubMed publications for compounds that could affect oxidative stress in chronic diseases. Our deep averaging network (DAN) approach highlighted twenty-five compounds. We filtered the results using a differential convolution network (DCN) to exclude less relevant drugs. We evaluated the ability of the compounds resulting from the previous phase to cross the blood–brain barrier and compared their physical and chemical properties. This approach pinpointed lipoxygenase inhibitor zileuton as a regulator of Nrf2 function. We validated our prediction by using Zileuton to activate Nrf2 in the macrophage cell line RAW26.7.

## 2. Results

We used a cutting edge approach to reduce the time of compound identification. Our RNA-seq analysis highlighted hypoxia as a major therapeutic target in LPS treated macrophages. We observed that Nrf2 levels, which is known to regulate response to reactive oxygen species in macrophages, is not upregulated in LPS treated macrophages. Activation of NRF2 could represent a potential polarizing M1 phenotype into an M2 anti-inflammatory phenotype [22]. Our drug repurposing strategy evolved around finding the best candidate to activate Nrf2 in RAW264.7 cells as a proof of concept. Our results point to zileuton as a putative Nrf2 modulator in depression.

### 2.1. RNA-seq Data Analysis

**Figure 1:**
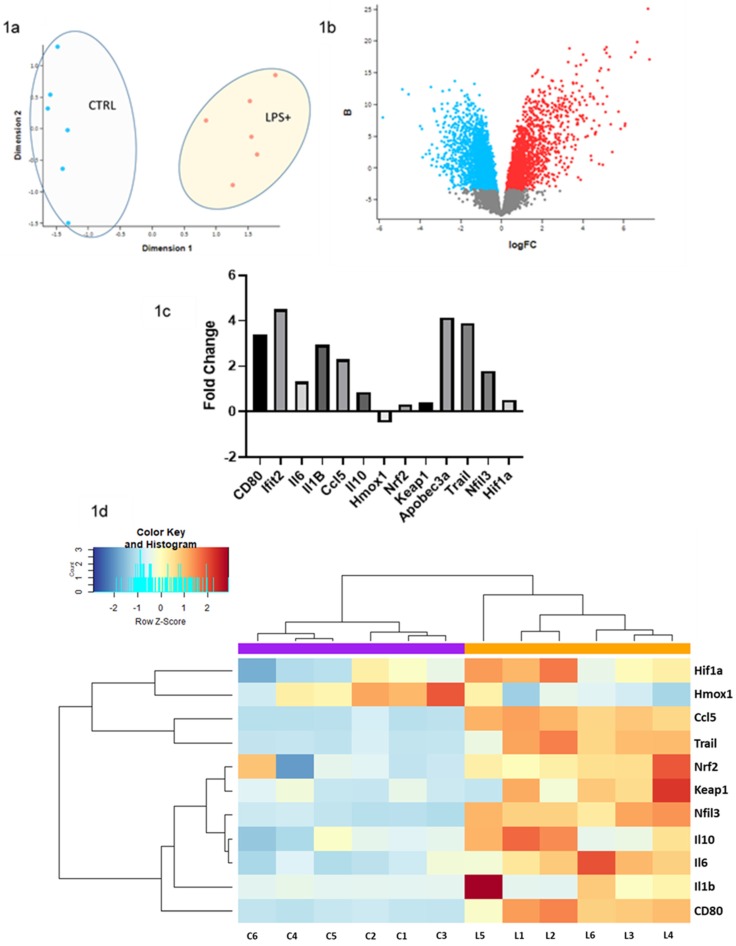

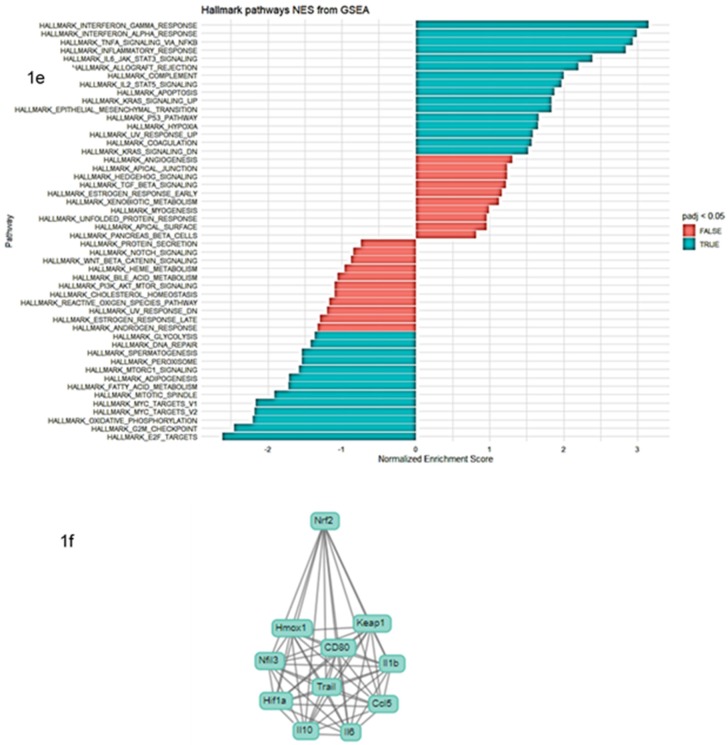
The hypoxia pathway is not activated in LPS treated macrophages. (**1a**) The PCA analysis indicates a clear separation between LPS treated cells versus controls. (**1b**), (**1c**) Many proinflammatory genes were upregulated in LPS+ cells while hypoxia related genes were not upregulated. (**1d**) Heat map of inflammatory pathway versus hypoxia and reactive oxygen species pathway. (**1e**) Pathway enrichment indicating that LPS+ cells have a proinflammatory profile. (**1f**) The gene analysis network shows that Nrf2 is putatively influencing all the chosen genes.

RNA analysis of the LPS treated macrophages versus not treated control macrophages showed an interlinking between the proinflammatory macrophages profile and hypoxia related genes. The two types of cells showed clear clustering using Principal component analysis (PCA) (Figure 1a), with upregulation of several markers of M1 macrophages profile such as Apobec3a [23], CD80, and Ifit2 with a fold change of 4.13, 3.4, and 4.5 respectively. Additionally, large group of genes with a proinflammatory function seems to be upregulated in the LPS treated macrophages (Figure 1b,c). Results indicate that in LPS treated macrophages, proinflammatory genes such as Il-1, Il-6, Ccl5, Il2ra, and Nlrc5 as well as Stat1 have increased with fold change of 2.9, 2.25, 2.3, 6.6, 1.83, and 1.44 compared to controls respectively (Figure 1c,d, Supplementary Table S4). Pathway enrichment analysis highlighted several immune associated hallmark pathways such as the IFNγ response, IFNα response, TNFα pathway, IL-6-STAT3 pathway, and IL-2–STAT5 pathway. It also highlighted a hypoxia pathway (Figure 1e). Surprisingly, few antihypoxia and anti-inflammatory genes such as Nfil3 [24,25] and Trail were also upregulated in LPS treated cells (fold change 1.8 and 3.9; Figure 1c). Paradoxically Hifn1a, Nrf2, Homx1, and Keap1 were not upregulated (Figure 1c). These observations lead us to hypothesize that manipulating Nrf2 pathway could prove critical for enhancing and supporting anti-inflammatory signature of macrophages during depression. Finally we confirmed that the proinflammatory response exhibited by the LPS treated macrophages could be linked to NRF2 pathway through applying GLASSO module using the webserver GeNeCK to analyze makers of both pathways (e.g., inflammatory and NRF2).

### 2.2. AI Embedding, Similarity, and Relevance Model Performance

**Figure 2:**
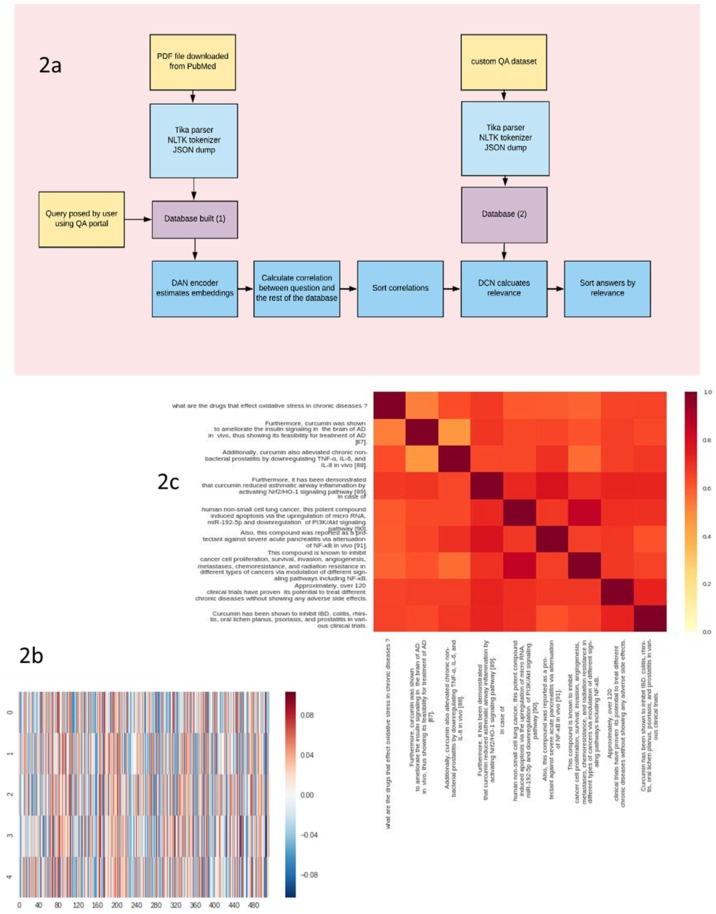

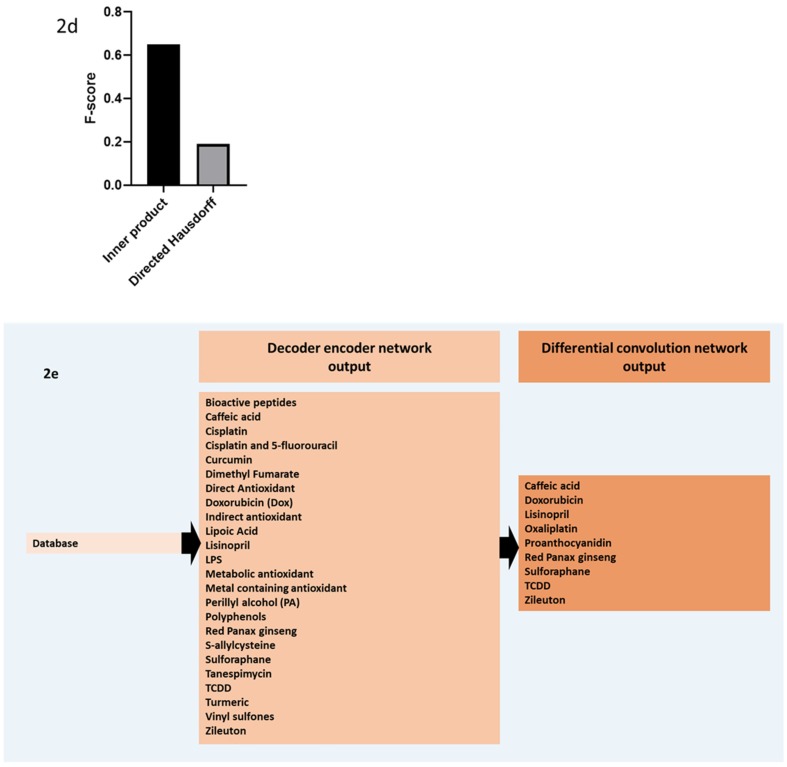
Artificial intelligence identifies 9 putative compounds as NRF2 activators. (**2a**) Deep neural network text mining workflow. (**2b**) Embedding is calculated using a deep averaging network (DAN) network (**2c**) Sentence similarity is calculated between the imposed question and each sentence and each paragraph of each document. (**2d**) We have chosen the inner product to score similarity because it has higher accuracy. (**2e**) We used a differential convolution network (DCN) network to extract features between each answer of the previous phase and the question. The DCN consist of a 2D layer and dense layer implemented in Keras library in python. After that, we used an mean squared error (MSE) approximation to calculate the distance between features of each answer and the imposed question.

Our deep learning network approach pinpointed nine putative drugs that could activate NRF2 pathway in macrophages (Figure 2a,e). The artificial intelligence (AI) workflow input was the question “what are the drugs that affect oxidative stress in chronic diseases?”. The workflow text mined 100 documents for relevant answers (Supplementary Table S1). Each sentence of every document as well as the imposed question was converted to a unique numerical vector using an AI encoder. Then, the embedding of each sentence was calculated (Figure 2b). After that, the sentence similarity score was computed (Figure 2c) using two different functions (inner product and Gromov–Hausdorff). We calculated the accuracy scores of the two similarity functions (Figure 2d). The inner product method f-score (0.67) was higher than that of the Gromov–Hausdorff (0.18). Thus inner product scores only were accepted. For every document, the answer was assigned to be the sentence with the highest score (Figure 2e and Supplementary Table S2). Finally, we employed a differential convolution network to compute the relevance between the answers sentences collected in the previous phase and the imposed question. We only accepted a threshold of 98% (Figure 2e).

### 2.3. Filtered Phase Results

**Figure 3:**
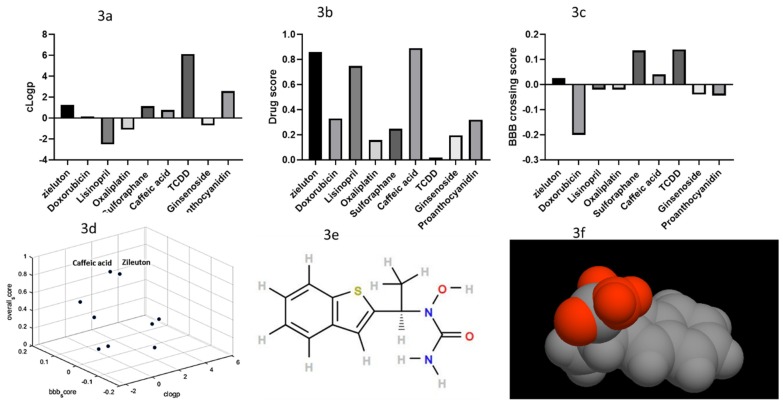
Physiochemical comparison of the resulting drugs. (**3a**) clogP calculation showing TCDD having high lipophilicity (logP > 5), which often contributes to high metabolic turnover, low solubility, and poor oral absorption while lisinopril is significantly soluble in aqueous solutions. (**3b**) Overall score of the drugs. (**3c**) Doxorubicin probability of crossing the BBB is very low. (**3d**) Overall comparison of filtered drugs showing zileuton and caffeic acid to be the most suitable compounds for further investigations. (**3e)** and (**3f**) Zileuton structure showing its biosuitability.

Our filtering phase identified two compounds that could activate Nrf2 in macrophages; caffeic acid and zileuton. We calculated clogP value for every compound resulting from the previous phase of investigation (Figure 3a). The clogP value of a compound is the logarithm of its partition coefficient between n-octanol and water log (coctanol/cwater). clogP is a classical measure of a compound’s hydrophilicity. Lisinopril, Oxaliplatin, and Ginsenodie have negative clogP values indicating high affinity for the aqueous phase. Thus these compounds are more likely to be soluble in aqueous solution with poor membrane permeability. On the other hand, low hydrophilicities and therefore high clogP values can cause poor absorption or permeation. It has been shown that the optimal clogP value was less than 5.0. TCDD (2,3,7,8-Tetrachlorodibenzo-p-dioxin) has clogP value of 6.12, while Zileuton value was 1.227. We also employed a drug score that takes into consideration Lipinski’s rule for oral absorption of compounds. According to that score, compounds must have a molecular weight lower than 500, the number of the hydrogen bond donor should be ≤ 5, and for the acceptor it should be ≤ 10. The topological polar surface area should be less than or equal to 140 and the number of rotatable bonds should be less than or equal to 10 and two violations of this rule would result to poor oral absorption (Figure 3b). Next we calculated blood–brain barrier passive diffusion ability for the nine compounds (Figure 3c). Only four compounds were found to be able to cross the blood-brain barrier (BBB), namely; sulforaphane, TCDD, zileuton, and caffeic acid. Overall, the two most probable candidates found by our workflow were caffeic acid and zileuton (Figure 3d). However, search of recently published literature indicated that caffeic acid has already been purposed to treat depression [26], so this compound was not investigated further. Our only candidate for experimental validation was found to be zileuton; (±)-1-(1-Benzo[b]thien-2-ylethyl)-1-hydroxyurea, which could inhibit 5-lipoxygenase among other function (Supplementary Table S2). Zileuton has −3.2 logS value, molecular weight 236, drug likeness of 2.2 with no risk of mutagenesis, tumorigenesis, irritating effects, or reproductive effects. It has mild side effects sinusitis (6.5%), nausea (5%), and pharyngolaryngeal pain and potential elevation of liver enzymes (in 2% of patients; Figure 3e,f, supplementary Table S3).

### 2.4. Experimental Validation Results

**Figure 4:**
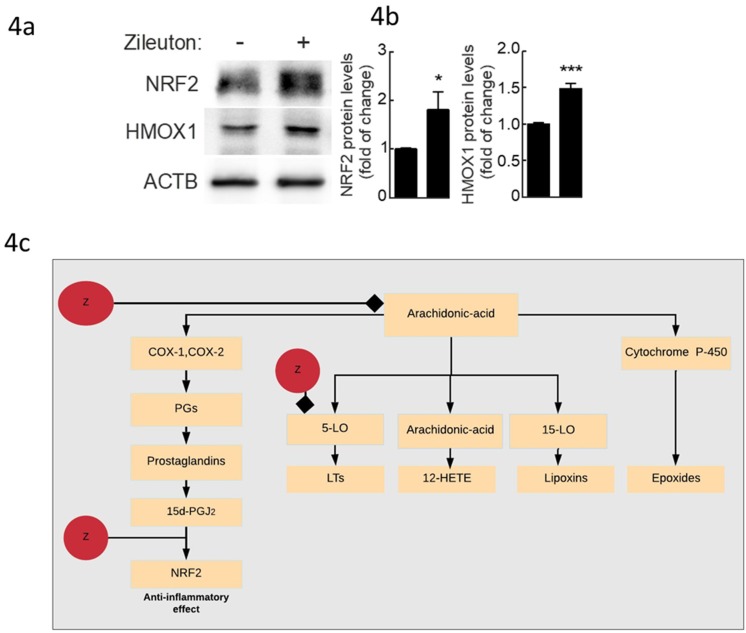
Zileuton activates Nrf2. A, Representative immunoblots for the indicated proteins in cell lysates from RAW264.7 cells treated with zileuton (10 µM, 16 h) in the absence of serum. (**4b**) Densitometric quantification of representative immunoblots from (**4a**) relative to ACTB protein levels. Data are mean ± SEM (*n* = 4). Statistical analysis was performed using Student’s t test. * *p* < 0.05; **** *p* < 0.001 vs. vehicle-treated cells. (**4c**) Zileuton model of action. In response to reactive oxygen species (ROS) stress, AA is released from membrane phopholipids by phospholipases. Free AA can be converted to bioactive eicosanoids through the cyclooxygenase (COX), lipoxygenase (LOX), or P-450 epoxygenase pathways. LOX enzymes (5-LO, 12-LO, and 15-LO) catalyze the formation of LTs, 12(S)hydroperoxyeicosatetraenoic acids and lipoxins (LXs), respectively. COX isozymes (constitutive COX-1 and inducible COX-2) catalyze the formation prostaglandin. The P-450 epoxygenase pathway catalyzes the formation of hydroxyeicosatetraenoic acids (HETEs) and epoxides. Zileuton was shown to inhibit 5-LO as well as prostaglandin production through suppressing prostaglandin biosynthesis by inhibition of arachidonic acid release in macrophages. Zileuton can also activate NRF2.

Zileuton increases NRF2 levels (Figure 4). RAW264.7 cells treated with zileuton (10 µM, 16 h) in the absence of serum showed increased NRF2 levels compared to vehicle-treated control cells. It is worth noting that we also observed an increase in HMOX1 protein levels. As expected, the increase in HOMOX1 was more significant than NRF2. Heme oxygenase-1 (HMOX1) is regulated by NRF2. Presumably, NRF2 levels increase first, and it has to translocate into the nucleus to induce HMOX1 transcription, which, then has to be translated.

## 3. Discussion

We focused on Nrf2 activation drug repurposing using an AI approach in Google Colab environment to regulate proinflammatory macrophages in depression. In biomedical applications, semantic similarity has become a valuable tool for analyzing the results in gene clustering, gene expression, and disease gene prioritization [2,3,27]. Our approach further extends these areas to make use of hundreds of drugs already approved for human usage. Our pipeline first calculates sentence embedding using a deep averaging network encoder. Then, we calculated sentence similarity between the posed question and the available dataset. Next we applied a DCN to filter less relevant targets. Our system identified zileuton as a putative compound to tackle neuroinflammation in depression. Interestingly, we predicted its ability to cross the blood–brain barrier by an in silico method. Moreover, we validated its ability to induce Nrf2 and its target Hmox1 levels in a macrophage cell line. Our approach seems capable of opening more opportunities for drugs repurposing for depression.

Our analysis of the Regan et al. RNA-seq data [21] pointed to a non-activated status of hypoxia associated genes such as Hifn1a, Nrf2, Homx1, and Keap1 (Figure 1c,d). This observation highlighted the suitability of Nrf2 as a potential drug target, in order to regulate inflammation response in depression (Figure 1c,d). Nrf2 pharmacological activation could play a vital role in regulating hypoxia and ROS in macrophages during depression. In depression, ROS are capable of producing membrane damage, changes in the inner proteins affecting their structure and function, lipids denaturation, and structural damage to DNA in the brain [28,29,30]. ROS also contributes to the gradual deterioration of macrophages functional characteristics in neurodegenerative diseases [31,32,33]. Oxidative imbalance produces reactive carbonyls that influence the ECM extracellular matrix environment of macrophages, decreasing their phagocytic activity towards apoptotic cells [34]. Furthermore, oxidative and carbonyl stress inhibits the activity of the transcriptional corepressor HDAC-2, which under normoxic conditions, helps to suppress proinflammatory gene expression [34]. The CNS is equipped with a repertoire of endogenous antioxidant enzymes, which are regulated by the transcription factor Nrf2 [35]. Under normal unstressed conditions, Nrf2 is bound to Keap1 [36]. Under environments of oxidative stress by either reactive electrophiles, toxins, or (antioxidant response element) ARE inducers, the interaction between Nrf2 and Keap1 is interrupted. Nrf2 translocates to the nucleus, where it binds to Smaf proteins [30]. This process increases the transcription rate of the antioxidant response elements [30]. Interestingly, Nrf2 was shown to be up-regulated in multiple sclerosis plaques and primarily expressed in macrophages [35]. Moreover, Nrf2 suppresses lipopolysaccharide-mediated macrophage inflammatory response by blocking IL-6 and IL-1β transcription, in Experimental autoimmune encephalomyelitis (EAE) mouse models [37]. It was suggested that the Keap1-Nrf2 system plays a key role in the stress resilience, which is involved in the pathophysiology of mood disorders. Remarkably, Nrf2 knock-out (KO) mice display a depression-like phenotype, and augmented serum levels of proinflammatory cytokines compared with wild-type mice [38]. It was also demonstrated that Nrf2-mediated antioxidant gene expression could reduce the macrophage M1 phenotype and ROS production [39]. Using Nrf2 activators has become a potential therapeutic strategy for numerous diseases [39,40]. However, the number of NRF2 activators applied in clinics is still small. Tecfidera (dimethyl fumarate), a potent Nrf2 activator, has been approved for the treatment of multiple sclerosis but long-term use of this drug can cause resistance and other side effects [40,41]. Nrf2 knockout mice with the anti-inflammatory drug rofecoxib reversed their depressive-like behavior [42]. Nardochinoid C was reported to inhibit inflammation and oxidative stress in lipopolysaccharide-stimulated macrophages. However, it is associated with acute renal failure [43]. Therefore, evidence indicates that the discovery of new and safer Nrf2 activators for clinical use in psychiatric disease has become an essential task in drug discovery [39].

Our system provides a context-aware approach for drug repurposing. The vast pharmacological knowledge available in the literature has made it increasingly feasible to employ text mining drug indications approaches. Swanson’s ABC links two concepts using a common relationship [44]. The clinically verified drug repurposing of fish oil to treat Raynaud’s syndrome was achieved using this approach [45]. However still, approaches based on concept co-occurrence within abstracts generate a high percentage of false positives hypotheses [46]. Mining databases such as the DrugBank could constitute a reasonable alternative, however many of the chemical compounds under current pharmacological research are not yet available through DrugBank. Another alternative is network-based approaches. DrugMap Central [47] is a network-based approach that uses information on chemical structures, drug targets, and signaling pathways to de novo alternative drug indications. However, this approach is time-consuming and also generates many hypotheses. Tari et al. [46] employed a parse tree, which is an ordered, rooted tree that represents the syntactic structure of a string according to a context-free grammar. In addition to being context-free, their method also is ontology-based. Our method, however, eliminates the need for ontology through a context-aware AI system. Using the inner products of the embedding vectors proved to be the most accurate approach (Figure 2d). There is a wide range of methods for calculating the similarity in meaning between two sentences including (i) baseline, (ii) word mover’s distance (iii) smooth inverse frequency, and (iv) AI encoders. The baseline method calculates the average of the word embedding of all words in the two sentences and then calculates the cosine between the resulting embedding [48]. This method lacks consistency as well as accuracy [48]. It also gives weight to irrelevant words [48]. Word mover’s distance measures the minimum distance that the words in one text need to “travel” to reach the words in the other text [49]. However, this method is slow. The smooth inverse frequency method tackles the problem of irrelevant words by using a weighted average of the word embedding [50]. It also removes common components by calculating the PCA for every sentence [50]. However, PCA is computationally complex and subject to random fluctuation. Here, we used a pretrained Google universal encoder, which has proven to be more accurate, less time consuming but more memory intensive [51]. In order to optimize its function, we added a correlation unit that takes the sentence embedding as its input and then calculates sentence similarity using different correlation algorithms. The correlation method that has the highest level accuracy seems to be the inner product approach. The reason behind could be that the Gromov–Hausdorff distance is essentially intractable as it involves the solution of an NP-hard optimization problem [52]. Thus, we employed the directed Hausdorff distance as an approximation to the Gromov–Hausdorff. However, the directed Hausdorff distance accuracy could be affected by the nonlinear nature of the embedded matrices. To increase relevance, we employed a custom deep learning network consisting of a dual convolution network and a difference function. We only accepted drugs with a relevance threshold of 98%. The DCN used extracts features from every sentence resulting from the previous phase and calculates a relevance score between every particular sentence and the imposed question. This step reduced the number of filtered drugs from 26 to 9 (Figure 2e).

We imposed strict drugs quality control based on three categories to remove drugs with lower suitability from our investigation. For example Lisinopril has high classical drug score (0.75), however, it seems to be hydrophilic with possible high solubility in aqueous solutions. This is mirrored in its low ability to diffuse through the BBB (Figure 3a–c). Similarly, TCDD has high ability to cross the BBB however its classical drug score is low. Sulforaphane (SFN), an isothiocyanate compound derived from broccoli, is a potent activator of the Nrf2 [53]. It has been suggested that supplementation of SFN-rich broccoli sprout could be prophylactic vegetable to prevent or minimize the relapse of inflammation in the remission state of depressed patients [53]. In agreement with these findings we found that sulforaphane has an optimal clogP and is capable of passing the BBB. However, its classical drug score was low (0.248). Therefore this compound needs to be furtherly developed in order to be more suitable to treat major depression patients. On the other end of the spectrum, oxaliplatin scored low on all three tests performed, achieving, 0.15, −0.02, and −1.1 in its drug score, BBB diffuse-ability, and clogP. In agreement with these findings, oxaliplatin was reported to increase cancer incidence especially in colorectal cancer patients [54]. Taken together, these findings highlight the importance of using filtering approaches to ensure the suitability of the results of the AI system.

Our research repurposes zileuton as anti-inflammatory enhancer by activating Nrf2 in macrophages. Oxidative stress is known to increase the levels of free arachidonic acid (AA) [55]. Free AA can be converted to bioactive eicosanoids through the cyclooxygenase (COX), lipoxygenase (LOX), or P-450 epoxygenase pathways (Figure 4c) [56]. LOX enzymes (5-LO, 12-LO, and 15-LO) catalyze the formation of LTs, 12(S)hydroperoxyeicosatetraenoic acids and lipoxins (LXs), respectively [57]. COX isozymes (constitutive COX-1 and inducible COX-2) catalyze the formation of PGH2 [58]. PGH2 is converted by cell-specific PG synthases to active prostanoids (including PGE2, PGF2a, PGI2, and TXA2) [57]. 5-lipoxygenase is found throughout the central nervous system, in both neuron and glia [59]. 5-lipoxygenase is also active mainly in myeloid cells, such as macrophages [60]. LTs are made predominately by inflammatory cells such as activated macrophages [61]. Zileuton is an active inhibitor of 5-lipoxygenase, and thus inhibits leukotrienes formation. Treatment with, zileuton, at an early stage of the development of the AD-like phenotype delays cognition impairments, reduces amyloid beta (Aβ) levels, and tau phosphorylation in mouse models of AD [62]. Zileuton treatment of AD-like phenotype of the 3xTg mouse model of AD starting at 12 months of age restored mice cognitive abilities and reduced Aβ deposition as well as tau phosphorylation [62]. It was also shown that blocking of 5-lipoxygenase with zileuton delayed the onset and reduced the cumulative severity of EAE mice [63]. Zileuton also possesses the ability to suppress prostaglandin biosynthesis by inhibition of arachidonic acid release in macrophages [64]. In confirmation with these studies [62], we observed the ability of zileuton to activate NRF2, probably through the prostaglandin pathway (Figure 4c). We also demonstrated an increase in the downstream target of *Nrf2*, *Hmox1*. *Hmox1* possesses anti-inflammatory properties through up-regulation of Il-10 and Il-1ra expression [65]. We speculate that zileuton could be inhibiting Keap1, either directly through acting as an electrophile, or indirectly through inhibiting 5-Lo or suppressing prostaglandin biosynthesis by inhibition of arachidonic acid release in macrophages. Overall, our results indicate that zileuton could be skewing macrophages polarization towards an M2-like phenotype [22,66]. Compared to known Nrf2 activators, zileuton characteristics including its ability to cross the BBB and biosuitability as well as its ability to decrease proinflammatory mechanisms in macrophages suggests it could have therapeutic applications in depression.

### Limitations and Future Development

Although our approach is innovative, it still suffers from several limitations. On the computational side, our method employed the Google recurrent network encoder using the transformer architecture. However, we have not compared its performance with other available encoders such as inferSent, which is a pretrained encoder that was developed by Facebook Research [67]. We did not apply any mathematical optimization techniques to identify the lowest possible Hausdorff distance [68]. The effect of changing zileuton concentration on Nrf2 expression levels is also yet to be investigated. It will also be interesting to validate the ability of zileuton to cross the BBB either by passive diffusion or active transport. Notably, although, immune activation with lipopolysaccharide (LPS) is known to produce a set of behavioral and cognitive alterations (anhedonia, anorexia, and memory deficits, among others) that resemble depression both in animals and in humans [69], recently it has been reported that M1 (= LPS+) macrophages are not equivalent to classically activated macrophages [70]. Thus in vivo validation is crucially needed. Additionally, investigating the effect of zileuton on activating Nrf2 pathway in microglia could also prove to be a critical therapeutic option in depression [71].

## 4. Methods

### 4.1. Analysis of RNA-seq Data

The overall design of the RNA experiment was as follows: primary human CD14+ monocytes were isolated from the whole blood of 6 donors (3 male and 3 female). These were transformed in macrophages through CSF-1 stimulation over a week. Cells were then subject to inflammatory stimulus with LPS (10 ng/mL) for 24 h or incubated for 24 h with no inflammatory stimulus [21]. LPS was used to induce an M1-like/proinflammatory phenotype. We downloaded non normalized expressions matrices from GSE85333. RNA-seq analysis was then performed in R using limma [72]. Briefly, we employed the limma RNA-seq differential gene expression method to compute the non-parametric approximations of mean–variance relationships. This allowed us to calculate the weights for a linear model analysis of log-transformed counts in conjunction with the empirical Bayes shrinkage of variance parameters. Differential expression analysis was performed to determine the differences in gene expression between +LPS cells and non-treated samples by fitting a linear model to compute the variability in the data with lmFit [72,73]. Pathway enrichment was done using the library fgsea [74]. The network between chosen genes was calculated using the GLASSO module utilizing the webserver GeNeCK [75] with default settings.

### 4.2. Building a Text Mining Deep Learning Neural Network

We used a question–answer pipeline consisting of two phases. The first phase aims to text mine relevant publications to extract compounds that could activate Nrf2. The sentence embedding was calculated for each publication using a DAN auto encode, together with the imposed question. All putative answers were ranked using an optimized sorter function, and the highest answer presented. The second phase aimed at filtering the answers according to their relevance using a DCN neural network.

### 4.3. The First Phase Used a DAN Neural Network Approach

For the DAN neural network testing, the database was built in the JSON format using publicly available literature. We downloaded 100 files in PDF format from PubMed with the query term “chronic diseases”, “drugs”, and “oxidative stress”. Text was then extracted from each of these files utilizing Tika python library (v 1.19) using the “parser.from_file” function to parse each file [76]. After that, the natural language processing (NLP) python module was employed to perform sentence tokenization into unique sentences with the tokenizer “nltk.data.load english.pickle” [77,78]. All tokenized sentences were then written into the database using json.dumps function in JSON module [79,80]. We employed the Google universal encoder to calculate sentence embedding in Tensorflow. Google universal encoder uses a deep averaging network (DAN) [81] as its composition function. The primary advantage of the DAN encoder is that compute time is linear to the length of the input sequence [6,19]. By this approach, input embedding for words and bi-grams are first averaged together and then passed through a feedforward deep neural network to calculate sentence embedding [18]. Overall, the encoder takes as input a lowercased Penn Treebank (PTB) tokenized string and outputs a 512-dimensional vector as the sentence embedding. The code was run in the Jupyter notebook on Google Colab [82,83]. We benchmarked several functions to identify the most accurate correlation technique. We analyzed the answers generated for ten manually benchmarked questions using the built database. The correlation between the questions embedding and each sentence embedding was calculated using two different approaches: (i) inner product and (ii) Gromov–Hausdorff. The results were then sorted and the highest answer was assumed to be the most relevant. Then, for each of the ten questions, the values of true positive, true negative, false positive, and false negative were calculated. The true positive is defined as the ability of the workflow to locate a sentence in the document that contains the name of a compound. Finally, we computed the f-score to evaluate our different correlation approaches.

### 4.4. The Second Phase Used a Differential Convolution Network (DCN)

To ensure the relevance of the putative compounds identified by the previous phase, we added a deep network that consists of two convolution networks, where the features of the embedding of the question sentence is extracted using a single convolution network. Similarly, the embedding of each sentence is fed into another convolution network. Then the similarity between the features is computed using the least mean square method. For training and testing this network, we used an in-house QA answer JSON dataset.

### 4.5. In Silico Prediction of Blood–Brain Barrier Diffusion

The ability to cross the blood–brain barrier (BBB) is the main obstacle facing neurodegenerative treatment drug development. To tackle this problem, we utilized BBB predictor (http://www.cbligand.org/BBB) to investigate the ability of all the nine compounds resulting from the AI investigation phase to cross the blood–brain barrier. First, the chemical structure was downloaded from DrugBank (https://www.drugbank.ca/drugs/DB00744) or PubChem (https://pubchem.ncbi.nlm.nih.gov/) in PDB format. Then, BBB predictor was employed using two different algorithms (i) support vector machine (SVM) and (ii) LiCABEDS. To ensure consistency, we employed four types of fingerprints (e.g., MACCS, Openbabel, Molprint 2D, and PubChem). Finally, the score of the BBB crossing probability was calculated using default settings.

### 4.6. Physicochemical Properties 

The physicochemical properties and the Lipinski’s rule of five parameters for prediction of oral bioavailability were calculated using molinspiration online program (www.molinspiration.com). clogP was determined by employing by Osiris Property Explorer (OPE; http://www.organic-chemistry.org/prog/peo/) [84]. Following Lipinski’s rule for oral absorption of compounds, we sorted the list of filtered drugs through calculating their molecular weight, number of hydrogen bond donor and acceptor, the topological polar surface area, and number of rotatable bonds, where two violations of this rule will result in the poor oral absorption.

### 4.7. Experimental Validation

RAW264.7 cells were grown in RPMI 1640 medium (GIBCO, Madrid, Spain, cat. no.11875093) supplemented with 10% fetal bovine serum FBS, cat. no. CH30160.03; Thermo Scientific, Hyclone, Logan, UT) and 80 μg/mL gentamicin (Laboratorios Normon, Madrid, Spain, 763011.1) Cells treated with zileuton (Sigma-Aldrich; Z4277, Madrid, Spain) in the absence of serum. After the indicated times, cells were lysed in lysis buffer (50 mM Tris-HCl pH 7.5, 400 mM NaCl, 1 mM EDTA, 1 mM EGTA, 1% SDS, 1 mM PMSF, and 1 µg/mL leupeptin), and samples were heated at 95 °C for 15 min, sonicated and precleared by centrifugation. Protein was quantified with DC™ Protein Assay (Bio-Rad, Hercules, CA. Primary antibodies were the following: anti-NRF2 (homemade; 1:5000), anti-HMOX1 (Enzo life sciences OSA110; 1:2000, Farmingdale, NY), anti-ACTB (Santa Cruz sc-1616; 1:5000), and anti-LAMINB (Santa Cruz sc-6217; 1:5000, Dallas, TX). Membranes were analyzed using the appropriate peroxidase-conjugated secondary antibodies (anti-mouse and anti-rabbit from GE Healthcare UK Limited, NA931V and NA934V, and anti-goat from Santa Cruz Dallas, TX, sc-2020). Proteins were detected by enhanced chemiluminescence (GE Healthcare, RPN2232).

## 5. Conclusion

Repurposing constitutes a rapid alternative to classical antidepressants. Our RNA-seq data analysis confirmed Nrf2 role as a main regulator of the pro vs. anti-inflammatory macrophages phenotype. Through our approach of applying the deep neural network to search and filter known drug compounds, we predicted the ability of zileuton to activate Nrf2 and its downstream targets. We validated our hypothesis in vitro. Further in vivo validation is crucial for determining the suitability of zileuton for clinical trials as an antidepressant drug. Additionally, our approach could naturally be applied to other depression disease models and drugs.

## 6. Patents

Zileuton for treating major depression disorder (Swedish Patent and Registration Office, pending).

## Supplementary Materials

The following are available online at https://www.mdpi.com/1420-3049/25/9/2155/s1. Table S1: A description of the database used in the manuscript. Table S2: Results of the first deep neural network. Table S3: Zileuton known targets. Table S4: Results of the RNAs-seq analysis.
