# Supplementary material for: Repurposing Zileuton as a Depression Drug Using an AI and In Vitro Approach
Source: Molecules. 2020 May 5;25(9):2155. doi: 10.3390/molecules25092155 (PMC7249014; doi:10.3390/molecules25092155)
Supplement: Supplementary file 1 [file molecules-25-02155-s001.zip › Table_2S_a.docx]

**List 1. Results of the question “what are the drugs that effect oxidative stress in chronic diseases?”.**

| **Detected sentence** | **Compound** |
| --- | --- |
| While usually less potent in their effects than synthetic pharmaceutical drugs,  these bioactive peptides are also less likely to accumulate in body tissues or to  confer serious side effects because nature has provided the mechanism for their  metabolism and utilization or excretion. | Bioactive peptides |
| The proglycation effect of caffeic acid leads to the elevation of oxidative stress  and inflammation in monocytes, macrophages and vascular endothelial cells. | Caffeic acid |
| It is interesting to note cis-diamminedichloroplatinum (cisplatin) as an example of  a drug that exhibits multiorgan toxicity with redox imbalance as a possible  mechanism. | Cisplatin |
| This effect was mediated by increased ROS production. Besides oxaliplatin,  autophagy inhibitors can enhance the effectiveness of other chemotherapeutic  drugs such as cisplatin and 5-fluorouracil in HCC. | Cisplatin and 5-fluorouracil |
| Effects of curcumin supplementation on exercise- induced oxidative stress in humans. | Curcumin |
| Control of Oxidative Stress and Inflammation in Sickle Cell Disease with the Nrf2  Activator Dimethyl Fumarate. | Dimethyl Fumarate |
| Direct Antioxidant Aryl amines and indoles-carotene, lycopene Polyenes-  carotene, lycopene, retinol Selenium containing compounds ebselen. Polyphenols-  Flavonoids, stilbenes, and hydroquinoneMonophenols: tocopherols (vitamin  E),estradiol (estrogen), 5-hydroxytryptamine (serotonin). | Direct Antioxidant |
| 18- Doxorubicin (Dox) is an anthracycline antibiotic used in numerous  chemotherapy regimens to treat haematological and solid tumours. | Doxorubicin (Dox) |
| Amino oxidase inhibitors, calcium antagonists,dopamine receptor agonists,  glutamate receptor antagonists, ion chelators, nitric oxide synthase inhibitors. | Indirect antioxidant |
| Lipoic acid as a potential therapy for chronic diseases associated with  Oxidative stress, | Lipoic Acid |
| These seemingly conflicting findings strongly suggest a very sensitive regulation  of a tight balance of positive effects of various ROS or RNS in LPS-stimulated  cytokine release, and inhibitory effects of the same molecules in the presence of  HEMA. | LPS |
| 1 N-acetyl-cysteine, glutathione, 2-oxothiazolidine-4-carboxylate, and other thioldelivering compounds N-butyl- -phenylnitrone. Carnitine, creatine, lipoic acid (thioctic acid),ubiquinone and idebenone. | Metabolic antioxidant |
| Manganese-containing mimetics of catalase/superoxide dismutase. | Metal containing  antioxidant |
| Based on previous data, we have used SH SY5Y cells as an in vitro model of PD ‐  to analyze the phytomedicinal potential of perillyl alcohol (PA), a monoterpenoid  obtained from essential oils of various plants such as sage, peppermint and  lavender. | Perillyl alcohol (PA) |
| A variety of polyphenols, mainly tannins (ellagitannins and proanthocyanidins),  which through in vitro, in vivo and clinical evidence have demonstrated a  potential to regulate and improve glucose homeostasis, attenuate dyslipidemia,  protect against inflammation, oxidative stress or prevent excessive weight gain. | Polyphenols |
| Implications of red Panax ginseng in oxidative stress associated chronic diseases. | Red Panax ginseng i |
| Lipid peroxidation, mitochondrial dysfunction and neurochemical and behavioural  deficits in different neurotoxic models: protective role of S-allylcysteine. | S-allylcysteine |
| In addition, sulforaphane is found to have a cytoprotective effect at lower doses  whereas higher doses are associated with apoptosis, anti-angiogenesis, antihistone  deacetylase activity and metastasis inhibition in cancer cells. | Sulforaphane |
| The inhibition of ACE-1 is a favoured strategy in treating hypertension [101] and  several synthetic ACE inhibitors (captopril, lisinopril, enalapril and fosinopril) are  thus used for this reason in the treatment of hypertension [102] Despite their  effectiveness, synthetic ACE inhibitors are responsible for a number of unpleasant  side effects such as development of a cough, loss of taste, renal impairment, and  angioneurotic oedema and as a consequence there has been a trend recently to  explore and develop more natural inhibitors of ACE activity. | Synthetic ACE  inhibitors |
| Tanespimycin (17-N-allylamino-17-demethoxygeldanamycin, 17-AAG) is a  derivative of the antibiotic geldanamycin and has been shown promise in the  treatment of some types of leukemia or solid kidney tumors. The drug binds to and  inhibits HSP90, and additionally, in combination with a class of drugs that inhibit the  mechanistic target of rapamycin (mTOR), affects the metabolism of GSH and TRX,  thus increasing the degree of oxidative stress | Tanespimycin |
| TCDD induces hepatic lipid accumulation (steatosis) and progression to steatohepatitis with fibrosi | TCDD |
| Oral supplementation of turmeric decreases proteinuria, hematuria, and systolic  blood pressure in patients suffering from relapsing or refractory lupus nephritis: a  randomized and placebo-controlled study. | Turmeric |
| Vinyl sulfones targeting Nrf2-mediated gene transcription intended for anti-  Parkinson drug design | Vinyl sulfones |
| Effects of zileuton, a new 5-lipoxygenase inhibitor, in experimentally induced colitis  in rats. | Zileuton |
